# Editorial: Editors' showcase: obstetrics and gynecology

**DOI:** 10.3389/fmed.2023.1308488

**Published:** 2023-11-02

**Authors:** Simcha Yagel, Sarah M. Cohen

**Affiliations:** Department of Obstetrics and Gynecology, Faculty of Medicine, Hadassah Medical Center, The Hebrew University of Jerusalem, Jerusalem, Israel

**Keywords:** reproductive medicine, ovarian pathologies, diabetes, prenatal diagnosis, ultrasound, obstetric outcome, hematopoietic cell transplantation, luteal phase support

We launched the *Editors' showcase: obstetrics and gynecology* to attract groundbreaking research articles and reviews for our section, Frontiers in Medicine/Obstetrics and Gynecology. Our invited authors and authors did not disappoint. We received many articles for the Editors' Showcase, 17 of which were ultimately published. They ranged over the whole breadth of OB/Gyn specialties, including Reproductive Medicine, Epidemiology, Endocrinology, Ultrasound and Prenatal Genetic Testing, Traditional Chinese Medicine, and Basic Research. All together the 17 publications attracted some 16,000 total views, over 15,000 article views, over 5,000 downloads and counting.

Six articles looked at topics in Reproductive Medicine, four examining various ovarian pathologies. Mu et al. reviewed Resistant Ovary Syndrome (ROS), a rare gynecological endocrine disorder impacting women's reproductive health. Patients with ROS display typical female sex characteristics and karyotype and a standard ovarian reserve, yet suffer from elevated gonadotropin and low estrogen levels, leading to primary or secondary amenorrhea. Despite numerous case reports over five decades, ROS's pathogenesis remains unclear, with no effective treatment strategies established. Their review collated all available ROS reports, summarizing its pathogenesis and treatment options, aiming to guide clinical management and set a foundation for future research.

Also focusing on the ovary, Bai and Wang discussed Premature Ovarian Failure (POF), a growing concern affecting women under 40, characterized by secondary amenorrhea and hormonal imbalances. Though hormone replacement therapy is advised, a comprehensive approach is vital for patient wellbeing. The core cause is linked to the depletion of the ovarian reserve due to issues with primordial follicle activation, emphasizing the need for pathway-based interventions.

Biernacka-Bartnik et al. investigated the threshold value for HOMA-IR to identify insulin resistance using the SHBG level in Caucasian women with polycystic ovary syndrome (PCOS). Using data from 854 women with PCOS, the study found that those with low SHBG levels (< 26.1 nmol/L) also had higher HOMA-IR values. The determined cut-off value for HOMA-IR indicating insulin resistance was ≥2.1, aligning with European standards for insulin resistance.

Su et al. studied the effects of hematopoietic cell transplantation (HCT) on reproductive functions in female survivors. Involving 55 females under 40, the study found 72.7% showed signs of premature ovarian insufficiency (POI) post-HCT. The likelihood of POI was influenced by age during transplantation, conditioning regimen, and blood disease type, with those aged 21–40 at HCT time being most affected.

Two articles addressed topics in optimizing IVF treatment. Wan et al. explored the optimal timing for frozen embryo transfer (FET) post-oocyte retrieval by comparing immediate FET (within 60 days of retrieval) to delayed FET (>60 days post-retrieval) through a retrospective analysis. Analyzing 5,549 patients, the study found no significant differences in various pregnancy outcomes, including live birth rates, between the two groups. Both immediate and delayed FET yielded comparable pregnancy results.

Jiang et al. devised an open-label, non-inferiority RCT to evaluate the necessity of luteal phase support (LPS) during natural cycle frozen-thawed embryo transfer (NC-FET), given the existing uncertainties about its importance in ART success. This study involving 1,010 ovulatory women will compare outcomes with and without LPS.

Five articles in the RT investigated a range of topics in Obstetrics. Wang et al. performed a meta-analysis of 41 articles from 1980 to 2021 to investigate the impact of pregnancy interval on maternal outcomes. Their review revealed that short interpregnancy intervals (IPI < 6 months) are associated with higher risks of preterm birth, low birth weight, and other adverse outcomes, but not gestational hypertension or diabetes. The study calls for further research to inform pregnancy guidelines for childbearing-aged women.

Zhou et al. explored the impact of insulin on severe hypertriglyceridaemia (HTG) during the third pregnancy trimester. Comparing insulin-treated women with a control group on a low-fat diet, the study revealed the insulin group had significantly reduced prenatal lipid levels, decreased complications like HTG-AP, and improved pregnancy outcomes, including lower neonatal weight and reduced intensive care unit (ICU) admissions.

Herzberg et al. explored the gynecological and fertility complications after conservative treatment for placenta accreta spectrum (PAS). In a study involving 134 women with conservatively managed PAS and 134 matched controls, women with PAS had a higher need for postpartum operative procedures. However, there was no significant difference in fertility outcomes between the two groups post-treatment.

Zhang et al. conducted a meta-analysis to assess the impact of small-angle episiotomy on postoperative recovery in primiparous women. Analyzing 25 RCTs with 6,366 cases, the study found that small-angle episiotomy significantly decreased incisional tearing, reduced suturing time, and minimized incisional bleeding. However, there was no notable difference in severe laceration rates. The technique can be beneficially applied in clinical settings, considering maternal and fetal conditions.

Liu et al.'s study investigated the influence of excessive gestational weight gain (GWG) before and after 28 weeks on the delivery mode among women attempting trial of labor after cesarean (TOLAC), considering pre-pregnancy BMI. Analyzing data from a Chinese hospital between 2016 and 2022, the study found that 71.1% of 512 women achieved vaginal birth. While no link was identified between excessive GWG before 28 weeks and vaginal birth after cesarean (VBAC) rates, excessive GWG after 28 weeks significantly decreased VBAC rates across all BMI categories, regardless of weight gain before 28 weeks.

Topics in Gynecology included a wide range of different topics within the specialty, including Chinese medicines, endometriosis, pelvic organ prolapse and cystocele. Zhong et al. conducted a Bayesian network meta-analysis comparing Chinese patent medicines combined with hormone replacement therapy (HRT) for treating premature ovarian failure (POF). Analyzing 64 randomized controlled trials with 5,675 participants, they found HRT combined with Chinese patent medicines, especially Zuogui pills, to be more effective than HRT alone. However, due to the low quality of included studies, further high-quality, multi-center trials are needed.

Wang et al. investigated the fallopian tube's potential role in the development of ovarian endometriosis by studying the expression of the folate receptor-alpha gene (FOLR1) and its protein (FRA). Analyzing 144 tissue samples, they found that FOLR1 was highly expressed in fallopian tube and ovarian endometriosis tissues, with significantly lower levels in endometrial samples. The results suggest that the fallopian tube may play a key role in ovarian endometriosis, and FRA expression could offer potential therapeutic targets.

Wu et al. reviewed the molecular mechanisms underlying pelvic organ prolapse (POP), a common gynecological issue in middle-aged and elderly women. Despite the high incidence and significant clinical impact, current treatments remain suboptimal. The study delves into the relationships between POP and factors like MMPs/TIMPs, cyclins, microRNAs, and oxidative stress. The goal is to identify precise biomarkers or molecular targets for better POP prevention, diagnosis, and treatment. Further research is essential for improved understanding and interventions.

Yin et al. examined factors affecting cystocele and its Green classification in 357 primiparous women using three-dimensional ultrasound. Results showed 242 had cystocele. Factors like body mass index (BMI) at delivery and prolonged second stage of labor were linked to cystocele and bladder abnormalities. The study suggests weight control during and post-pregnancy and minimizing the second stage of labor to reduce cystocele risk.

Two papers focused on the importance of genetic diagnosis, both prenatally and in a pregnant patient. Kang et al. presented two cases highlighting inconsistencies between non-invasive prenatal testing (NIPT) and invasive testing for trisomy 21. Despite NIPT's advancements, invasive tests remain essential because placental DNA doesn't fully represent fetal genetic information. They emphasized that positive NIPT results need confirmation through invasive testing, and even negative NIPT results necessitate continuous monitoring due to potential placental mosaicism.

Huang et al. presented the first case of a pregnant woman with TSC2/PKD1 contiguous gene deletion syndrome, a rare condition combining tuberous sclerosis and polycystic kidney disease symptoms. The patient exhibited multiple clinical signs, including renal cysts and angiomyolipoma. There was an observed increase in the size of renal abnormalities during pregnancy. Enhanced monitoring and prenatal genetic testing can ensure optimal outcomes for both mother and fetus.

## Author contributions

SY: Conceptualization, Writing—original draft, Writing—review & editing. SC: Conceptualization, Writing—original draft, Writing—review & editing.

